# Diagnostic value and key features of computed tomography in Coronavirus Disease 2019

**DOI:** 10.1080/22221751.2020.1750307

**Published:** 2020-04-23

**Authors:** Bingjie Li, Xin Li, Yaxuan Wang, Yikai Han, Yidi Wang, Chen Wang, Guorui Zhang, Jianjun Jin, Hongxia Jia, Feifei Fan, Wang Ma, Hong Liu, Yue Zhou

**Affiliations:** a Department of Oncology, The First Affiliated Hospital of Zhengzhou University, Zhengzhou, People’s Republic of China; b Department of Respiratory Medicine, The First Affiliated Hospital of Zhengzhou University, Zhengzhou, People’s Republic of China; c Department of Radiology, The First Affiliated Hospital of Zhengzhou University, Zhengzhou, People’s Republic of China

**Keywords:** Coronavirus Disease 2019, computed tomography, diagnosis, SARS-CoV-2, ground-glass opacity

## Abstract

On 31 December 2019, a severe acute respiratory syndrome coronavirus 2 (SARS-CoV-2) emerged in Wuhan, Hubei province, China, and caused the outbreak of the Coronavirus Disease 2019 (COVID-19). To date, computed tomography (CT) findings have been recommended as major evidence for the clinical diagnosis of COVID-19 in Hubei, China. This review focuses on the imaging characteristics and changes throughout the disease course in patients with COVID-19 in order to provide some help for clinicians. Typical CT findings included bilateral ground-glass opacity, pulmonary consolidation, and prominent distribution in the posterior and peripheral parts of the lungs. This review also provides a comparison between COVID-19 and other diseases that have similar CT findings. Since most patients with COVID-19 infection share typical imaging features, radiological examinations have an irreplaceable role in screening, diagnosis and monitoring treatment effects in clinical practice.

## Introduction

On 31 December Wuhan, Hubei province 2019, a series of unidentified pneumonia disease cases were reported in, central China, with epidemiological links to the Huanan Seafood Wholesale Market [1]. Soon after, the Chinese Centre for Disease Control and Prevention isolated a novel coronavirus from human epithelial cells as the causative agent of this outbreak, which was named severe acute respiratory syndrome coronavirus 2 (SARS-CoV-2) [2]. On 30 January 2020, the World Health Organization (WHO) declared the transmission of SARS-CoV-2 as the sixth Public Health Emergency of International Concern [3]. On 12 February 2020, WHO named the disease caused by SARS-CoV-2 as Coronavirus Disease 2019 (COVID-19). On 11 March 2020, WHO formally announced that COVID-19 was a pandemic [4]. As of 18 March 2020, there are now more than 194,000 confirmed cases in 164 countries, with 7864 cases of mortality. The number of confirmed cases and deaths exceeds those of Severe Acute Respiratory Syndrome (SARS) and the Middle East Respiratory Syndrome (MERS). At present, the basic reproductive number of SARS-CoV-2 has been estimated to lie between 2.2 and 3.9 by different teams in China and other countries [5–9]. Unlike SARS and MERS, asymptomatic COVID-19 patients have been reported to be contagious [10]. Therefore, the transmission capability of SARS-CoV-2 may be greater than that of SARS. Although the fatality rate of COVID-19 is lower than SARS and MERS, COVID-19 has killed more people than SARS and MERS combined [11].

Sequence analysis shows that SARS-CoV-2 has the typical genome structure of the coronavirus and belongs to the beta coronavirus genus, which is the seventh member of the family of coronaviruses that infect humans [12–14]. COVID-19 is the third widespread pandemic virus-related after SARS in 2002 and MERS in 2012. SARS-CoV-2 has an 80% similarity to SARS and 50% similarity to MERS at the genome level [12]. SARS-COV-2 can be easily transmitted from person to person through close contact, droplets and aerosols [5,6,15,16]. According to recent reports, the most common symptom of SARS-COV-2 infection is fever, cough, myalgia and fatigue, and the less common symptoms were sputum production, haemoptysis, and diarrhoea[17,18]. The median incubation period is 5.1 days [19]. Therefore, early diagnosis and isolation are important to control the spread of the epidemic. At present, real-time reverse-transcription–polymerase-chain-reaction (RT–PCR) assay for COVID-19 has been developed and is being used in clinics. In the current emergency, the high false negative rate and the shortage of RT–PCR implies that many COVID-19 patients may not be identified on time. However, most patients with COVID-19 has been diagnosed with pneumonia and characteristic computed tomography (CT) imaging patterns; thus radiological examinations are essential for the early diagnosis and evaluation of the disease. In this review, we focus on the imaging characteristics and changes in patients with COVID-19 to provide some help for frontline clinicians.

## Typical imaging features of COVID-19

Currently, chest radiography remains the first-line imaging test for identifying pneumonia because it offers simplicity, low cost, and considerable information, and can be considered the reference standard [20]. However, COVID-19 is similar to SARS and MERS with regard to clinical manifestations and imaging features. Considering the experiences with SARS and MERS treatments, CT is more sensitive and specific than X-rays and can identify the abnormalities in the lungs earlier [21,22]. In addition, 5 patients underwent chest radiography along with chest CT examinations in a recent study. Of these, 2 patients had normal chest radiography findings, despite also having CT examinations performed on the same day that showed ground-glass opacity (GGO). Moreover, the chest radiography findings of the other 3 patients did not show the peripheral predominance that was visible on their respective CT scans [23]. The reason for the negative chest radiography findings in patients with COVID-19 might be twofold. First, chest radiography is not sensitive for the detection of GGO which may not have been of sufficient density to be seen on radiographs. Moreover, these faint opacities initially within the basal and retrocardiac location of the lungs may have been difficult to see as they may have been obscured by the overlying diaphragm in the frontal view and by mediastinal structures in the lateral view. Therefore, chest radiography is not recommended as the first-line imaging modality for COVID-19. However, chest radiography is valuable in the treatment of COVID-19. The systematic quantitative evaluation of sequential chest radiographs in severely ill patients can be used to dynamically monitor the illness state of patients when CT is not available or when severely ill patients cannot be moved. In summary, chest radiography could be normal in a patient with COVID-19 even after the onset and progression of typical clinical symptoms. CT has a higher sensitivity than chest radiography with abnormalities in the lungs being identified earlier. Thus, CT may be used as an initial investigative tool in patients with high clinical suspicion for COVID-19.

So far, only seven case series [18,23–28] and some case reports [29–37] have investigated the chest CT imaging features of COVID-19 pneumonia. According to these reports, patients with COVID-19 can show typical imaging features at the early stages of the disease. Chest CT plays an important role in the screening and preliminary diagnosis of COVID-19 pneumonia. Among the first 41 COVID-19 patients that were laboratory-confirmed in Wuhan before January 2, a limited analysis of chest imaging showed all patients had abnormalities in chest CT, and 40 (98%) of them had bilateral lung involvement. In this study, it was found that patients with severe cases who were on admission were more likely to have bilateral multiple lobular and subsegmental areas of consolidation, while admitted patients with mild cases were more likely to have bilateral GGO and subsegmental areas of consolidation [18]. Similar characteristics were found in another 51-person study cohort: most pulmonary lesions involved bilateral lungs with multiple lung lobes, and were mainly distributed in the posterior and peripheral part of the lungs. Moreover, 39(77%) patients had pure GGO on CT, 38(75%) had GGO with reticular and/or interlobular septal thickening on CT, 30 (59%) had GGO with consolidation on CT scans, while 28(55%) had pure consolidation on CT scans. Notably, consolidation is considered a sign of disease progression. Younger patients tended to have more GGO, while older patients tended to show more pulmonary consolidation. This is also consistent with the phenomenon where the prognosis of young patients is better than that of elderly patients [24]. Chung et al. found that the crazy-paving pattern often appeared in patients with COVID-19 in addition to the above-mentioned characteristics, while lung cavitation, discrete pulmonary nodules, pleural effusions, and lymphadenopathy were absent [25]. One study reviewed the recent literature to synthesize the findings of 233 patients infected with COVID-19, and all studies showed consistent findings, with GGO and consolidation being the most common findings on CT [23]. In other reports, CT images of these patients showed the same typical imaging feature [29–37]. It is not difficult to see that the CT of patients with COVID-19 has special characteristics, with the predominant features outlined as follows: (1) single or multiple GGO, which is mainly a subpleural distribution; (2) crazy paving; (3) patchy GGO with segmental pulmonary consolidation; (4) pulmonary consolidation [38,39].

COVID-19 pneumonia has nonspecific and diverse chest CT imaging features, which are closely related to pathology. By investigating the pathological characteristics of patients who died from COVID-19, bilateral diffuse alveolar damage (DAD) with cellular fibromyxoid exudates were noted, indicating the acute respiratory distress syndrome [40]. The pathological features of COVID-19 are extremely similar to those of SARS and MERS [41,42]. The main lung pathology in SARS and MERS is DAD in various stages, which is basically the histological prototype of acute lung injury [43,44]. According to the previous pathological mechanism of SARS, the emergence of GGO suggests that SARS-CoV-2 causes airspace exudates, and pauci-inflammatory alveolar and interstitial oedema [45]. Crazy paving appearance consists of GGO with superimposed interlobular and intralobular septal thickening, reflecting interstitial lesions [46]. Bilateral extensive consolidation of the lungs results from a combination of the large number of desquamated and exudate cells and protein exudates that congest the lung tissues, and the extensive formation of hyaline membranes in the alveoli. Pulmonary nodules are not the typical imaging features of COVID-19. However, Li et al. reported an interesting case where a young woman with COVID-19 had a single right upper lobe nodule with the halo sign [47]. The halo sign can be present in viral infections and organizing pneumonia, however, the main pathological drivers of this sign in COVID-19 are unknown [48]. More research is needed to elucidate the histopathological findings of this emerging viral infection so that various radiological observations can be better understood.

## Imaging features at different stages

The typical imaging characteristics of patients with COVID-19 have different manifestations at different stages of the disease. We can evaluate the disease severity of COVID-19 and the efficacy of treatment through dynamic observation of CT imaging to guide clinical management. GGO is the most typical imaging feature of COVID-19 [49]. In a retrospective study, the analysis of the CT images of 21 patients showed that most patients had single or multiple GGOs in the early stages of the disease, and the scope of GGOs continued to expand with disease progression. In the later stages of COVID-19, GGO is often combined with other imaging features, such as pulmonary consolidation, crazy paving appearance, etc.[27]. In the current case reports, the CT images of these patients showed the same pattern of change [26,29,31–33,35,37]. One of the most representative cases is the CT change in a 44-year-old transportation staff of the Huanan seafood market in Wuhan. At the time of admission, bilateral multiple GGOs appeared on the subpleural region of the lungs, and as the disease progressed, CT showed crazy paving appearance, and the number and range of GGOs gradually expanded to the entire lung [29]. We can speculate that at the early stages of the disease, single or multiple GGO is the most common symptom, mostly distributed unilaterally or bilaterally in the posterior aspects and periphery of the lungs, with bilateral distribution being more common. During the progression of the disease, the number and range of GGOs gradual expanding, and fusion and crazy paving appearance may occur in some cases. In the later stages of the disease, GGOs present a diffuse distribution of bilateral lungs.

Pulmonary consolidation is also one of the characteristics of CT in patients with COVID-19, which is considered as a sign of disease progression [24]. Pan et al. found that pulmonary consolidation is rare in the early stages of COVID-19. With the progression of the disease, pulmonary consolidation gradually appears, and the range of lesions continues to expand. In the later stages of COVID-19, the range of pulmonary consolidation becomes larger and diffuse [27]. This pattern is clearly shown in current case reports of some patients with COVID-19 [26,29,31,37]. Particularly, in a study by Song et al., the CT images of a 75-year-old man at admission clearly showed the absence of pulmonary consolidation, while CT images on day 3 after admission showed more consolidations [24]. In addition, in the case report of a 32-year-old man, as the condition improved, the pulmonary consolidation on the patient’s CT image gradually disappeared [32]. According to these reports, larger consolidation indicated disease progression, while absorption and smaller size of these lesions indicated improvement [50–52]. We speculate that at the early stages of the disease, pulmonary consolidation is rare. During the progression of the disease, pulmonary consolidation begins to appear and gradually becomes the main imaging feature. In the later stages of the disease, the range of pulmonary consolidations is more extensive, and some severe cases even show a “white lung” appearance.

## Diagnosis and differential diagnosis

According to the current diagnostic criteria, nucleic acid testing is the gold standard for the diagnosis of COVID-19 [53]. However, the current nucleic acid test is time-consuming, and may yield false-negative results due to laboratory error or insufficient viral material in the specimen [54,55]. Current reports show that in some cases, patients may exhibit typical imaging features but may have multiple negative results of RT–PCR tests of nasopharyngeal or throat swabs [56]. Xie et al. found that 3% (5/167)of patients had initially negative RT–PCR findings but positive chest CT, and finally, both RT–PCR and chest CT were consistent with COVID-19 [57]. Fang et al. compared the sensitivity of initial chest CT and RT–PCR for COVID-19, and the detection rate for initial CT (98%) was higher than that for first RT–PCR (71%) (*P* < 0.001) [58]. On the basis of these findings, Ai et al. conducted out another study, they performed multiple RT–PCR tests and chest CT tests on 1014 suspected COVID-19 cases. Overall, 88% (888/1014) of patients had positive chest CT scans, whilst 59% (601/1014) of patients had positivity RT–PCR. Importantly, as many as 93% of all patients whose RT–PCR became positive after an initially negative test result had CT features suggestive of COVID-19 [59]. Therefore, in the context of a typical clinical presentation, detailed exposure and travel history, patients with CT features should be highly suspected to have COVID-19 despite negative nucleic acid test results. In these cases, repeat swab testing and patient isolation should be considered. Furthermore, a normal chest CT scan does not exclude the diagnosis of COVID-19 [36].

Of note, COVID-19 is not only similar to SARS and MERS in clinical manifestations, but also in imaging features. The disease processes are similar insofar as GGO and consolidation are the primary findings on CT scans [60,61]. This should be expected as the three infectious agents are part of the coronavirus family, and viruses in the same viral family share a similar pathogenesis [62]. However, there are some differences in their imaging findings. For patients with SARS, GGOs with consolidations are the main findings, while unifocal involvement is more common than multifocal or bilateral involvement on CT [63]. Although angiotensin-converting enzyme 2 (ACE2) is a potential receptor of SARS-COV and SARS-COV-2, different affinities of spike protein and ACE2 receptors may be one of the reasons for the difference [64]. Regarding MERS, there is a tendency for a basilar and subpleural distribution. Moreover, the presence of pleural effusion and pneumothorax can be considered an important predictor of a poor outcome; these features are rare in SARS and COVID-19. Moreover, MERS progresses more rapidly to respiratory failure than SARS and COVID-19 [65]. In addition to SARS and MERS, other viral pneumonia, non-viral pneumonia, and other non-infectious diseases, such as influenza pneumonia, respiratory syncytial virus pneumonia, mycoplasmal pneumonia, etc., are differential diagnoses that should be considered [66–74]. Table 1 lists typical imaging characteristics and clinical features of the pneumonias mentioned above.

**Table 1.** Imaging characteristics and clinical features of common causes of pneumonia similar to COVID-19 pneumonia.

Diseases	High-risk groups	Clinical symptoms	CT imaging findings
**COVID-19**	Elderly people; People with comorbidities	Fever Cough Myalgia or fatigue Headache Dyspnoea	Single or multiple GGOs with subpleural distribution Crazy paving Diffuse consolidation with GGO
*Other viral pneumonia*			
Influenza pneumonia	Elderly people; Children under 5 years old	Stuffy nose Runny noses Sore throat Dry cough	Small patch GGOs and consolidation with subpleural and or peribronchial distribution Bilateral reticulonodular areas of opacity
RSV pneumonia	Children under 2 years old	Cough Stuffy nose High fever	An airway-centric distribution, with areas of tree-in-bud opacity and bronchial wall thickening With or without consolidation along the bronchovascular bundles
Rhinovirus pneumonia	Children	Stuffy nose Runny noses Sore throat	Multifocal GGO and interlobular septal thickening
Adenovirus pneumonia	Children under 2 years old	Fever Cough Dyspnoea Drowsiness	Bilateral multifocal GGO with patchy consolidations Bronchopneumonia that resembles bacterial pneumonia (lobar or segmental distribution)
SARS pneumonia	Young and middle-aged people	Fever with chills Dyspnoea Diarrhea Cough Headache	Subpleural GGO and consolidation prominent lower lobe involvement interlobular septal and intralobular septal thickening Unifocal involvement is more common than multifocal or bilateral involvement.
MERS pneumonia	Children; Elderly people; People with comorbidities	Fever with chill Cough Shortness of breath	Extensive GGO and occasional septal thickening and subpleural effusion Bilateral, basilar and subpleural airspace
*Non-viral infectious pneumonia*			
Mycoplasmal pneumonia	Children	Headache Fever Myalgia or fatigue	Flabellate shadows extended outward from the hilus pulmonis
*Non-infectious pneumonia*			
Hypersensitivity pneumonia	An exposure history of inhaled antigen	Fever Dry cough Shortness of breath Chest pain	Extensive, bilateral, and symmetric GGO Centrilobular nodules
Pulmonary alveolar proteinosis	Young and middle-aged people	Shortness of breath after activity Cough Expectoration	GGO sharply demarcated from surrounding normal lung tissue, which created a geographic pattern.
Interstitial pneumonia	Middle-aged and elderly people	Shortness of breath after activity Cough	Ground-glass attenuation Broad honeycombing in a predominantly peripheral distribution

GGO: ground-glass opacity; RSV: respiratory syncytial virus; SARS: Severe Acute Respiratory Syndrome; MERS: Middle East Respiratory Syndrome.

## Conclusion

This review, we summarized the CT imaging characteristics of COVID-19, mainly GGO and pulmonary consolidation, with prominent distribution in the posterior and peripheral part of the lungs. Through the analysis of imaging at different stages of COVID-19, we can speculate that the appearance and exacerbation of pulmonary consolidation signs may be related to disease progression and the diagnostic value of patients’ prognosis. Although positive nucleic acid testing is still the gold standard diagnosis, with regard to the typical clinical diagnosis, Wuhan exposure or close contact history, CT features can be used for the clinical diagnosis of COVID-19 infection despite negative nucleic acid test results. CT is very sensitive for COVID-19 lesions, and it currently has an irreplaceable role in screening, diagnosis and monitoring treatment effects in clinical practice.
